# Tight Shoes

**Published:** 1876-01

**Authors:** 


### TIGHT SHOES

Interfere with the pleasure of locomotion, cause corns, and even rheumatic gout; hence it is worth while to repeat what we have formerly recommended as an infallible and easy method of having a new foot-covering fit as easily as an old shoe—just put on two pairs of thick stockings before the measure is taken or before fitting your feet with ready-made shoes; then when you get home pull off both pair, put on one thin pair, wear them for a few days and then put on thicker. This simple expedient will prevent an incalculable amount of discomfort, irritation and loss, in one year.

### CLEANING SHOES.

To do this easily, harmlessly and well in winter, is worth knowing. Scrape off the mud or wet dirt with an old spoon handle, or which is better, a wooden knife, then with a soft, damp rag or sponge remove what the knife failed to do, then set them back from the fire for five or six hours, or more; they will then take a polish as easily as before they were wetted; in this way they can be cleaned without scarcely soiling the fingers at all, and a great deal of extra brushing will be saved.

Boots and shoes for the winter should be large enough to admit of cork soles which, if taken out every night and dried well, will keep the feet warm all the time, without which condition no person can possibly have good health, while there are many whose only obstacle to good health is cold feet.

The feet are so far from the centre of the system that the circulation in them is easily checked and then disease begins; hence it is of great importance that persons in going to their place of business, with the expectation of remainin g in several hours, should pull off their tight-fitting boots and put on a pair of easy fitting slippers or shoes; and they will find that on putting on their boots again at night to go home, it is done with considerable difficulty. This is because the feet have swollen during the day, a natural result from the blood and other fluids accumulating in them, partly from their being in a standing position for a considerable portion of the time; and partly from the unrestrained condition of the foot, the circulation is more free and healthful; but if a tight boot is kept on all day, it becomes more and more compressed every hour, and by night the circulation is almost arrested, the feet are cold and clammy and damp, and this soon becomes their constant condition, instead of a few hours towards the close of the day; but this very change to a loose slipper or old shoe, on arriving at the shop, or store, or office, will in a very short time be followed by lameness, or stiff joints, or a cold, impregnating the whole system unless the slippers or shoes are first made very warm. Common-sense points out the fact that harm must result from changing a loose, cold shoe for a warm one.

A fruitful cause of colds is the wearing during the winter while out of doors, boots or shoes with thinner soles, even if the weather is milder. When a thick soled shoe is put on in the early part of the winter, it should be used until the first of May, or at least until the winter is broken up. In the effort to keep the feet warm the experience of one man is no safe guide to another. Some keep their feet warm during the coldest weather by wearing cotton stockings; others are more successful by wearing woolen hose. The only rational plan is for each one to experiment on himself and observe the result closely. Others again succeed best by wearing two pairs of hose at the same time, one of woolen, the other of cotton; these differences arise from the fact that the circulation of some is more vigorous than that of others; some are on their feet all the time; others sit almost all day.

